# RE: Dipstick Spot urine pH does not accurately represent 24 hour urine PH measured by an electrode: Omar M, Sarkissian C, Jianbo L, Calle J, Monga M, Int Braz J Urol. 2016;42:546-9

**DOI:** 10.1590/S1677-5538.IBJU.2016.0370

**Published:** 2016

**Authors:** Viroj Wiwanitkit

**Affiliations:** 1 Suvannhabhumi Clinical Training, Research and Development Center, Institute of Natural Medicine Science Development and Establishment Project, Surindra Rajabhat University, Surin Province, Thailand

*To the editor,*

Dear Editor, the recent report on “Dipstick Spot urine pH does not accurately represent 24-hour urine pH measured by an electrode” is very interesting (1). Similar to the previous report by Kwong et al., the diagnostic property of urine dipstick is the issue for consideration. In fact, it is no doubt that the urine dip stick cannot be comparative to automated electrode urine chemistry analyzer. However, the usefulness of dipstick is still noted and should be mentioned. First, the use of dipstick is fit for field work where the advanced technology is not available. To help the accuracy, the use of semi-automated urine strip reader might be a solution for the problem of naked eye urine strip reading. Nevertheless, it should be noted that the urine dip stick can be only a screening tool. The confirmation is noted. As noted by Wockenfus et al. “therapy protocols should not alternate between dipstick and pH meter urine pH monitoring (3).”
